# Nodal Peripheral T-Cell Lymphoma: Therapeutic Challenges and Future Perspectives

**DOI:** 10.3390/cancers17071134

**Published:** 2025-03-28

**Authors:** Ho Pui Jeff Lam, Faisal Amin, Suzanne O. Arulogun, Mary Gleeson

**Affiliations:** Department of Clinical Haematology, Guy’s and St Thomas’ NHS Foundation Trust, London SE1 9RT, UK; faisal.amin@gstt.nhs.uk (F.A.); suzanne.arulogun@gstt.nhs.uk (S.O.A.); mary.gleeson@gstt.nhs.uk (M.G.)

**Keywords:** peripheral T-cell lymphoma, brentuximab vedotin, nodal T follicular helper cell lymphoma, anaplastic large cell lymphoma, histone deacetylase inhibitors

## Abstract

Nodal peripheral T-cell lymphomas (PTCLs) comprise a heterogeneous group of diseases, with common subtypes including nodal T follicular helper cell lymphoma (nTFHL), anaplastic large cell lymphoma (ALCL), and peripheral T-cell lymphoma, not otherwise specified (PTCL-NOS). These subtypes are characterised by distinct phenotypes and pathobiologies. Historically, CHOP chemotherapy has been the standard frontline treatment, though recent advancements, particularly the introduction of brentuximab vedotin in the Echelon-2 study, have significantly improved outcomes. Despite these advances, relapsed and refractory PTCLs remain challenging to treat due to limited effective second-line therapies. Ongoing research is focused on developing novel agents targeting key oncogenic drivers and mutations. These drugs include histone deacetylase inhibitors, hypomethylating agents, PI3-kinase pathway inhibitors, dihydrofolate reductase inhibitors, and JAK/STAT pathway inhibitors, each showing varying efficacy across PTCL subtypes. In addition, combination therapies are being explored for potential synergistic effects. Cellular therapies, including chimeric antigen receptor T-cell (CAR-T) therapy, are also emerging as promising treatment strategies for this difficult-to-treat group of lymphomas. This review provides an updated overview of the current therapeutic landscape of PTCLs, a group of malignancies traditionally associated with poor prognosis.

## Simple Summary

This review article provides a comprehensive summary of the therapeutics for nodal peripheral T-cell lymphomas (PTCLs), examining past treatments, current strategies, and emerging therapies in the pipeline, highlighting the evolving nature of this field.

## 1. Introduction

Peripheral T-cell lymphomas (PTCLs) present a significant clinical challenge despite recent advances in the development of novel therapeutic agents, guided by a deeper understanding of the pathobiology and the genetic and molecular characteristics underlying this complex and heterogeneous group of aggressive non-Hodgkin lymphomas (NHLs). Originating from mature T- and natural killer (NK-) cells and comprising more than 30 distinct subtypes broadly categorised into nodal, extranodal, leukaemic, and cutaneous forms, these lymphomas are rare and make up less than 15% of all NHLs globally [1]. The clinical presentation of PTCLs is diverse, differing considerably between subtypes, and may include systemic B symptoms, generalised lymphadenopathy, organomegaly, as well as various extranodal manifestations, with the skin and gastrointestinal tract being the most commonly affected sites. While there is considerable geographic and ethnic variation in the prevalence of the disease, PTCLs primarily affect adults. The incidence steadily increases with age, and the median age at diagnosis is between 60 and 65 years, with the exception of anaplastic lymphoma kinase (ALK)-positive anaplastic large cell lymphoma (ALCL), which is more common in children. Overall, there is a male predominance, with the male-to-female ratio ranging from 1.5:1 to 3:1, depending on the subtype [1,2,3].

## 2. Nodal Peripheral T-Cell Lymphomas

PTCL nosology has evolved over time, with the latest proposals put forward in the World Health Organization Classification of Haematolymphoid Tumours (WHO-HAEM5) and the International Consensus Classification (ICC) of Mature Lymphoid Neoplasms, both published in 2022 [4,5].

Nodal T-cell lymphomas are more prevalent in Western countries, accounting for 60–70% of all mature T- and NK-cell lymphomas [6,7], and will be the focus of exploration in this review. The two classifications largely align in their descriptions within the nodal subtype, with the exception of the ICC considering follicular helper T-cell lymphoma (TFH lymphoma) as a single entity, whereas the WHO categorises this into nodal TFH cell lymphoma angioimmunoblastic type, follicular type, and not otherwise specified (NOS). The most recent editions of both now also acknowledge Epstein–Barr virus (EBV)-positive nodal T-and NK-cell lymphoma, with the ICC introducing the provisional entity *primary nodal EBV+ T cell/NK cell lymphoma*, distinct from PTCL-NOS.

Nodal T follicular helper (TFH) cell lymphοma (nTFHL) is the most common subtype (25–40% of PTCLs), followed by PTCL, not otherwise specified (PTCL-NOS; 20–27% of PTCLs), and systemic ALCL (6–25% of PTCLs) [8]. ALCL is further subdivided into ALK-positive and ALK-negative subtypes. EBV-positive nodal T- and NK-cell lymphoma is less common, predominantly occurring in Asia in association with immunodeficient conditions, including human immunodeficiency virus infection. Breast implant-associated anaplastic large cell lymphoma (BIA-ALCL) is a rare entity, specifically linked to malignancy arising in the periprosthetic space surrounding breast implants.

Most nodal PTCLs are diagnosed at an advanced stage of the disease, are clinically aggressive, and carry a poor prognosis. The average 5-year overall survival (OS) rate is between 30 and 40% [9]. Prognostication is commonly determined by the International Prognostic Index (IPI) Score. Risk factors for poor outcome include advanced age, stage III-IV disease, >1 extranodal site, performance status ≥2, and elevated lactate dehydrogenase (LDH) [10]. Other T-cell NHL-specific scoring systems have been applied and include the Prognostic Index for PTCL, unspecified (PIT), the International peripheral T cell lymphoma Project score (IPTCLP) in PTCL-NOS, and the Angioimmunoblastic T-cell lymphoma (AITL) prognostic score in nodal TFH lymphoma, angioimmunoblastic type [11,12]. Additional features suggested as predictors of poor outcome include a high Ki-67, positivity for EBV, the presence of systemic B symptoms, low albumin, and mediastinal lymphadenopathy. Furthermore, in ALCL, ALK-negative status and high beta-2 microglobulin are associated with poorer outcomes [13].

## 3. Pathobiology

New insights into the molecular and genetic landscape of nodal PTCL, along with an enhanced understanding of the mechanisms driving T-cell oncogenesis, have been essential for refining the classification of each distinct entity in the current classification systems. These contribute to our existing knowledge of the disease’s histopathological characteristics and phenotypic patterns, as well as its shared clinicopathological features (Table 1).

nTFHLs encompass three distinct morphological subtypes: angioimmunoblastic type, follicular type, and not otherwise specified (NOS). This category of PTCLs is united by a common immunophenotype, primarily the expression of two or more TFH cell markers, including CD10, BCL6, CXCL13, PD1, and ICOS [4]. The angioimmunoblastic type is the most prevalent and is characterised by a tumour microenvironment rich in inflammatory cells, which include follicular dendritic cells, alongside a network of arborising vasculature. There is usually complete effacement of the nodal architecture (pattern type III), although partial effacement is also observed (pattern types I and II). In contrast, the follicular type predominantly manifests within the follicles, while the NOS subtype is generally located in the paracortex and between follicles. Genomic analyses of these neoplasms have revealed characteristic mutations in epigenetic modifiers: TET2, DNMT3A, and IDH2. Additionally, alterations in the proximal T-cell receptor (TCR) signalling pathway, frequently driven by mutations in RHOA, are a common feature [14,15,16].

ALCL is characterised by the presence of large pleomorphic lymphoid cells exhibiting strong expression of CD30. Systemic ALCL is divided into ALK-positive and ALK-negative ALCL based on the presence or absence of anaplastic lymphoma kinase expression. Microscopically, there are typically cohesive sheets of neoplastic cells with an intrasinusoidal growth pattern within lymph nodes [17,18]. Uniquely, ALK-positive ALCL has the most favourable prognosis among the nodal PTCLs, with 5-year OS exceeding 70% in adults and higher in children [1]. Aberrant expression of the ALK protein occurs due to gene rearrangements involving the ALK gene, with more than 20 potential partner genes reported. It is most commonly associated with the t(2;5)(p23;q35) chromosomal translocation leading to the NPM-ALK fusion protein, which exhibits constitutive tyrosine kinase activity involving various signalling pathways involved in oncogenesis, such as JAK-STAT3, RAS-ERK, and PI3K-AKT. These signalling cascades promote cell survival, proliferation, and immune evasion, providing ALK-positive ALCL multiple targets for potential therapeutic intervention. Less common gene mutations identified in this category include NOTCH1 and TP53, with the latter related to a poorer outcome. ALK-negative ALCL is genetically more complex despite being morphologically indistinguishable from ALK-positive ALCL. Approximately 20–30% of cases exhibit DUSP22 gene rearrangements at 6p25.3, leading to the overexpression of LEF-1, a transcription factor involved in immune regulation and cellular differentiation. This was first described by Feldman et al. [19] and has traditionally been associated with a more favourable prognosis, akin to that observed in ALK-positive ALCLs, potentially obviating the need for consolidative therapy [20]. However, this concept remains debated, with some studies failing to demonstrate a clear outcome benefit [21,22], while its prognostic significance has been questioned in others [23]. DUSP22 rearrangements are often mutually exclusive with TP63 inv(3)(q26q28), observed in 5% of ALK-negative ALCL cases, which correlates with a more aggressive disease course and poorer prognosis [24,25].

PTCL-NOS is typically a diagnosis of exclusion for mature T-cell NHLs that do not meet the criteria for other defined subtypes, and consequently, it is histologically and genomically more diverse. Gene expression profiling (GEP) has identified two major molecular subgroups: PTCL-TBX21 (50–60%) and PTCL-GATA3 (30–40%) [25]. TBX21 is a master regulator of Th1 cells and is associated with mutations in epigenetic regulators that enhance NF-kB signalling, which confers a more favourable prognosis. However, a subset of PTCL-NOS within this group with a more cytotoxic phenotype and poorer outcomes are noted. GATA3 is a master regulator of Th2 cells and is characterised by more complex genomic alterations, including mutations in key tumour suppressor genes such as TP53, CDKN2A, and PTEN. As such, it is typically associated with a less favourable outcome.

## 4. Management and Therapeutics

### 4.1. CHOP

For many years, combination chemotherapy with CHOP (cyclophosphamide, doxorubicin, vincristine, and prednisolone) has been the gold-standard treatment for nodal peripheral T-cell lymphomas, based on the findings of the landmark SWOG phase III study [26]. This study demonstrated a complete response (CR) rate of 44% and a partial response (PR) rate of 36% in patients with undifferentiated aggressive lymphomas, alongside a 3-year overall survival (OS) rate of 54%. Subsequent prospective clinical trials have further elucidated the efficacy of CHOP in the challenging nodal T-cell disease group. A phase II study by Reimer et al. in 2009, which assessed CHOP induction followed by autologous stem cell transplant (autoSCT), reported an overall response rate (ORR) of 79% and a CR rate of 39%, with 3-year progression-free survival (PFS) and OS rates of 36% and 48%, respectively [27].

### 4.2. Attempts to Improve on CHOP

Historically, increasing efficacy in PTCL treatments often led to higher toxicity, limiting potential benefits. This challenge persisted until the introduction of brentuximab, which has shifted this dynamic. For instance, a randomised phase II study by the UK group compared a gemcitabine-based regimen, GEM-P (gemcitabine, cisplatin, and methylprednisolone), to CHOP. The study was prematurely closed due to the lack of significant improvement in CR rates with GEM-P [28]. Similarly, the Southwest Oncology Group evaluated another gemcitabine-based regimen, PEGS (cisplatin, etoposide, gemcitabine, and methylprednisolone), which yielded disappointing results, with an ORR of 39% and a 2-year PFS of only 12% [29]. A phase III randomised controlled trial conducted by the Groupe Ouest Est D’Etude des Leucémies et Autres Maladies du Sang (GOELAMS) compared a VIP-reinforced ABVD regimen (etoposide, ifosfamide, cisplatin alternating with doxorubicin, bleomycin, vinblastine, and dacarbazine) to CHOP. However, no significant difference in event-free survival (EFS) was observed between the two regimens, with a 3-year EFS of 35% in the VIP-ABVD arm versus 26% in the CHOP arm [30].

In 2010, the German High-Grade Non-Hodgkin Lymphoma Study Group (DSHNHL) explored the addition of etoposide to the CHOP regimen in T-cell lymphoma subtypes [31]. This modification improved the 3-year event-free survival (EFS) to 75.4%, compared to 51.0% in the standard CHOP arm in younger adult patients (up to age 60) with a normal LDH level. However, no OS benefit was observed. When evaluating individual disease subtypes, the improvement in EFS was only significant in the ALK-positive ALCL subgroup, with a 3-year EFS of 91% versus 57%. Modest improvements were also seen in other T-cell subtypes with a low IPI (≤1), though these did not reach statistical significance. These benefits were not apparent in patients older than 60, likely due to increased toxicity.

Efforts to improve clinical outcomes have built upon the established CHOP backbone in a number of studies. Alemtuzumab, an anti-CD52 humanised monoclonal antibody, was investigated in combination with CHOP14 in the phase III ACT1 (aged 18–65 years) and ACT2 (aged 61–80 years) trials, respectively [32,33]. The ACT1 study allowed for consolidative autoSCT by design. At three years, PFS was 37% in ACT1 and 28% in ACT2, while OS was 52% and 37%, respectively. No significant improvements in PFS or OS were observed with the addition of alemtuzumab, and concerns regarding increased toxicity in the experimental arms were raised.

Bevacizumab, an anti-angiogenic humanised monoclonal antibody targeting VEGF, was studied in combination with standard CHOP therapy in the phase II E2404 trial [34]. The results were suboptimal, with 1-year and 3-year PFS rates of 44% and 16%, respectively. Furthermore, concerns regarding the potential cardiotoxicity associated with the concurrent use of bevacizumab and anthracycline-based therapy emerged during the study.

More recently, lenalidomide, an immunomodulatory agent, was explored in combination with CHOP for AITL in the REVAIL study [35]. The primary endpoint of improving complete metabolic response (CMR) was not achieved (41%; 95% CI 30–52.7%). Molecular sub-analysis revealed that the DNMT3A mutation was associated with a lower CMR rate and significantly shorter PFS. Romidepsin, a histone deacetylase inhibitor, was investigated in combination with CHOP in a randomised phase III trial conducted by the LYSA group for PTCL, excluding ALK-positive ALCL [36]. The study failed to demonstrate any improvement in ORR (63% vs. 60.5%), PFS (2-year PFS 43% vs. 36%, *p* = 0.0962), and OS (2-year OS 64% vs. 63%, *p* = 0.477). Consequently, romidepsin was voluntarily withdrawn from its indication for relapsed or refractory PTCL following its previous fast-track approval by the U.S. Food and Drug Administration (FDA) in 2012.

### 4.3. Brentuximab Vedotin

The next major advancement in PTCL treatment was driven by brentuximab vedotin, an antibody–drug conjugate targeting CD30. Initially evaluated in the relapsed/refractory setting for ALCL, it demonstrated an impressive ORR of 86%, with 57% of patients achieving a CR [37]. A 5-year follow-up study reported an OS rate of 79% and a PFS rate of 57%. [38] Building on this success, the global phase 3 randomised Echelon-2 study evaluated the combination of brentuximab vedotin (A+CHP) with chemotherapy compared to standard CHOP therapy in frontline PTCL treatment [39]. The study found a median PFS of 48.2 months in the A+CHP group, compared to 20.8 months in the CHOP group. A recent 5-year follow-up update from this phase III study continued to show a clinically significant improvement in OS for the A+CHP group (70.1% vs. 61.5%), corresponding to a 28% reduction in the risk of death [40]. Common side effects include peripheral neuropathy, which occurred in 52% of patients in the A+CHP arm compared to 55% in the CHOP arm. A grade 3 event was observed in one patient, with the majority showing improvement during subsequent follow-up [40]. The Echelon-2 study led to the regulatory approval of brentuximab vedotin for use in ALCL, reflecting the high enrolment of patients with systemic ALCL. However, the trial was not sufficiently powered to provide robust data on non-ALCL histologic subtypes, limiting its applicability across the broader PTCL spectrum and its licencing. Further exploration into the efficacy of brentuximab vedotin in PTCL was provided by Herrera et al. in a phase II study involving CHEP-BV followed by brentuximab consolidation. The study demonstrated very good ORR approaching 94% in ALCL and 90% in non-ALCL. This translated to 18-month PFS of 81% in ALCL and 49% in non-ALCL subgroups [41].

CD30 expression is a hallmark feature of ALCL but varies across other subtypes of T-cell lymphomas [18]. In the Echelon-2 study, local laboratory immunohistochemistry (IHC) was used to assess CD30 expression, with eligibility criteria requiring a minimum expression of ≥10%. The SGN35-032 phase II study further explored the efficacy of brentuximab in non-ALCL T-cell lymphomas with less than 10% CD30 expression, categorising patients into two subgroups: CD30 < 1% and CD30 1–10%. The overall and complete response rates were comparable across both subgroups, with an ORR of 79% and a CR rate of 66% in the CD30 < 1% group, and an ORR of 78% and CR of 67% in the CD30 1–10% group [42]. Similarly, Herrera et al. reported comparable ORR and CR rates in their CHEP-BV cohort, with 93% and 67% in the CD30 1–9% group, and 90% and 87% in the CD30 > 10% group, respectively [41]. These findings suggest that brentuximab vedotin maintains its efficacy regardless of the quantitative level of CD30 expression. Regulatory approval for brentuximab did not specify a CD30 cut-off.

### 4.4. Transplant Consolidation

Autologous stem cell transplantation (autoSCT) has become a widely accepted consolidation therapy for patients with nodal PTCL in first complete remission (CR1), and is incorporated into international guidelines, including those from the National Comprehensive Cancer Network (NCCN) and the clinical practice recommendations of the American Society for Blood and Marrow Transplantation, recognising the short duration of remission with chemotherapy alone [43]. However, this approach is primarily based on a limited number of prospective and retrospective studies, many of which lack randomisation or controlled groups. Furthermore, only a minority of patients may ultimately be suitable for autologous transplant consolidation, with only 25–36% proceeding to autoSCT in randomised studies [28,43].

Among the earlier cohorts, Corradini et al. investigated the role of autoSCT consolidation, including patients with ALK-positive ALCL. As expected, patients with ALK-positive ALCL exhibited the best outcomes, with a 12-year OS rate of 62% and a 12-year EFS rate of 54%. These results align with existing survival data for ALK-positive ALCL patients receiving the best available therapies (5-year OS of 70–90%). Generally, autoSCT is often considered unnecessary for this subgroup, and ALK-positive ALCL patients are typically excluded from studies evaluating the benefit of transplant consolidation. However, in the absence of robust data, upfront transplantation may still have a role in selected ALK-positive patients with high IPI (≥4) and aggressive presentations, given the 5-year OS rate as low as 33% and failure-free survival (FFS) of 25% in this high-risk category. This approach should be considered on an individual basis. [1]

Some of the larger cohorts such as the Nordic Lymphoma Group (NLG) reported on a cohort of 166 PTCL patients in 2012, of whom 155 underwent autoSCT following induction therapy if either CR or PR was achieved. The 5-year OS and PFS rates for this cohort were 51% and 44%, respectively [44]. A prospective German study by Wilhelm et al. in 2016 examined 111 PTCL patients, 75 of whom proceeded to receive autoSCT. The estimated 5-year OS rate for those undergoing autologous stem cell transplantation was 57%, compared to just 23% for those who did not receive the procedure [45]. Additionally, the Swedish Lymphoma Registry identified 252 patients with nodal PTCL and enteropathy-associated T-cell lymphoma, demonstrating superior OS (hazard ratio [HR] 0.58, *p* = 0.004) and PFS (HR 0.56, *p* = 0.002) in patients who underwent auto-SCT compared to those who did not in a multivariate analysis [9]. In contrast, Fossard et al. conducted a study across 14 centres in Europe involving 269 patients and found no survival advantage associated with autoSCT as a consolidation treatment after induction [46].

The prospective multicentre study COMPLETE directly compared survival outcomes with or without autoSCT consolidation in those who achieved CR1. The study demonstrated superior survival for advanced-stage disease or intermediate to high IPI scores, and particularly in the AITL disease group [47]. The Geltamo/Fil study, which recently added to the body of literature, analysed 174 patients in CR1, as opposed to the intention-to-treat model, reporting that frontline autoSCT consolidation was beneficial in 5-year PFS (63% vs. 49%; *p* = 0.042). While overall survival did not show a significant difference in univariate analysis, autoSCT was predictive of longer OS in the multivariate Cox regression model (HR 0.57, *p* = 0.048) [48].

In terms of allogeneic stem cell transplant (alloSCT), Schmitz and colleagues’ study found higher relapse rates in autograft patients (36% vs. 0%) but no transplant-related mortality in the autograft group in a randomised phase III study. In contrast, alloSCT had a higher transplant-related mortality (31%) but no relapses, suggesting alloSCT should be reserved for selected patients, particularly those who relapse after autoSCT [49].

In conclusion, despite the varying results across studies, autologous stem cell transplantation remains widely endorsed as a consolidation approach for fit PTCL patients who do not have ALK-positive ALCL and are in CR1. A randomised controlled trial is essential to definitively confirm the benefit of autograft.

### 4.5. Drugs and Targets in Development

Advancements in the genetic and molecular characterisation of this complex disease have been crucial for the development and integration of novel targeted therapies into existing treatment strategies, both in the frontline and relapsed/refractory setting of PTCLs. Table 2 summarises the potential drug targets and the main new and emerging therapeutic options that have recently been approved and are currently being studied.

Epigenetic modifying agents have emerged to represent a more targeted approach to treat nodal PTCLs, particularly in nTFHLs, which frequently harbour mutations in epigenetic regulators. Histone deacetylase inhibitors (HDACis) are one such class of agents that can induce changes in the epigenome by modulating chromatin and influencing transcription. Romidepsin was evaluated in a single-arm phase II trial for relapsed or refractory (R/R) patients before its accelerated approval in 2012 [50]. The trial reported an ORR of 25% and CR rate of 15%. A modest increase in the trend of response was observed in the AITL subtype, with an ORR of 30% and CR rate of 19%. Following the aforementioned negative phase III study comparing Ro-CHOP to standard CHOP therapy [36], the drug’s approval was voluntarily withdrawn. Noteworthily, an exploratory analysis of the TFH lymphoma subtype in this phase III study identified a significant prolongation in PFS in the Ro-CHOP arm (19.5 months vs. 10.6 months). Belinostat, a pan-HDAC inhibitor, was approved for relapsed or refractory PTCL in 2014. The phase II BELIEF (CLN-19) study demonstrated an ORR of 25.8%, with 10.8% achieving CR and 15% achieving PR [51]. Again, AITL ORR was modestly better at 45.5%. Notably, when combined with CHOP, the ORR increased significantly to 86%, with a CR rate of 71%, showing promising results in the frontline setting [64]. Ghione et al. analysed 127 patients with AITL or PTCL-NOS treated with HDACi at relapse and found increased response rates in nTFHLs compared to PTCL-NOS (ORR 56.5% vs. 29.4%) [65]. TFH phenotype was a significant predictor of response by logistic regression. PFS showed a trend in favour of the TFH phenotype but this did not reach significance.

Pralatrexate, a folate antagonist, was also evaluated in PTCL treatment. In the phase II PROPEL study for R/R PTCL patients, pralatrexate showed an ORR of 29% and a CR rate of 11% [57]. In combination with CHOP, the regimen achieved an ORR of 83.9%, with a CR rate of 66% [66]. Furthermore, pralatrexate was tested in combination with CEOP therapy and autoSCT in the T-cell Consortium trial [67]. Despite promising results, the 2-year PFS and OS rates of 39% and 60%, respectively, did not surpass the historical outcomes observed with standard CHOP therapy. Currently, the CRESCENDO: SPI-BEL-301 phase 3 study is underway to compare the efficacy of Belinostat combined with CHOP versus pralatrexate combined with COP, in relation to standard CHOP therapy. This study aims to clarify the relative efficacy of these two agents in the frontline treatment of PTCL (NCT06072131).

Azacitidine, a hypomethylating agent, was initially explored in a retrospective study involving 12 patients with AITL, which demonstrated promising results with an ORR of 75% and a CR rate of 50% [68]. The ORACLE phase III study randomised 86 patients with R/R AITL or nFHTL to receive either oral azacitidine or the investigator’s choice of standard therapy [69]. The study reported a median PFS of 5.6 months in the azacitidine arm, compared to 2.8 months in the standard arm. However, the CR rate at 3 months was lower in the azacitidine group (11.9%) compared to the standard treatment arm (22.7%). The study did not meet its primary endpoint of an improvement in PFS. Nevertheless, these observations have laid the groundwork for further investigation into the potential of azacitidine in combination with other therapeutic agents. A phase II study of oral azacitidine plus CHOP as initial treatment for PTCL reported ORR of 75% and CR of 75% [70]. Most recently, a 5-year update showed PFS of 26.3% and OS of 61.1%, compared to 2-year PFS and OS of 66% and 69%, respectively, suggesting high risks of relapses despite achieving remission [71]. Currently, azacitidine is also being studied in combination with romidepsin in a phase II trial (NCT04747236) and with chidamide, another class I histone deacetylase (HDAC) inhibitor recently approved by the China Food and Drug Administration, in another phase II study (NCT04480125).

Among the approved therapies for PTCL, crizotinib is a tyrosine kinase inhibitor that specifically targets ALK. This small molecule gained FDA approval in 2021 for use in paediatric patients aged 1 to 21 years with R/R ALK-positive ALCL. The approval followed the results of the multicentre, single-arm ADVL0912 (CRISP) study [72], which demonstrated an ORR of 88% and a CR rate of 81%. Further evaluation of crizotinib in combination with chemotherapy (according to the ALCL99 protocol) was conducted in paediatric patients with newly diagnosed ALK-positive ALCL. In the ANHL12P1 trial, this combination resulted in a CR rate of 92% after two cycles, with a 2-year PFS rate of 77% and an OS rate of 95% [73]. Additionally, the study incorporated a brentuximab arm alongside standard chemotherapy. Given the high incidence of thromboembolic events observed in the crizotinib arm, and the comparable efficacy between the two treatment regimens, the authors concluded that crizotinib is unlikely to offer superior benefits over brentuximab. Later generations of ALK inhibitors, such as brigatinib, ceritinib, alectinib, and lorlatinib, have demonstrated significant efficacy in the treatment of non-small cell lung cancer, by substantially prolonging PFS and overcoming resistance mechanisms [74,75,76,77]. Their roles and efficacy in PTCL have shown promise in a limited cohort of patients [78,79] and are currently being further assessed in ongoing trials (NCT05770037/NCT03505554).

The P13K kinase pathway inhibitor, duvelisib, was assessed in the PRIMO study for patients with R/R PTCL. The study reported an ORR of 48% and a CR rate of 33% [55,80]. In addition, the frontline use of duvelisib is currently being investigated in the ongoing Alliance study (NCT04803201). This trial is evaluating the combination of duvelisib with CHOP chemotherapy for patients aged over 60, and with CHOEP chemotherapy for those under 60 [81].

Another significant therapeutic target in PTCL is the JAK/STAT pathway. The well-known JAK1/2 inhibitor, ruxolitinib, has been evaluated, with an observed clinical benefit rate (CBR; i.e., response lasting > 6 months) of 35%, in a study including both PTCL and cutaneous T-cell lymphoma (CTCL) [81]. Those exhibiting JAK/STAT pathway activation in PTCL demonstrated CBR of up to 53%. It was also revealed that those with documented JAK2 mutations or ≥30% phosphor-STAT3 staining by IHC were more likely to achieve a response. Golidocitinib, a selective JAK1 inhibitor, has been assessed in the phase 2 JACKPOT8 study, demonstrating an ORR of 44.3%, including a 24% CR rate [59].

Ultimately, as nodal PTCLs are a heterogeneous group of malignancies with distinct pathogenesis, treatment responses are influenced by underlying genetic abnormalities. In light of this, the current treatment paradigm is shifting toward combination therapies (Table 3). This evolving strategy aims to address the limitations of single-agent treatments by leveraging the synergistic effects of multiple therapeutic modalities. Moreover, these approaches are essential for evaluating the tolerability of combined toxicities, a critical consideration for optimising patient outcomes.

### 4.6. Immune Therapy

Chimeric antigen receptor T-cell (CAR-T) therapies have demonstrated significant success in treating B-cell lymphomas [94,95]. However, their application in T-cell disorders is still emerging and faces several technical challenges. These challenges primarily arise from the shared expression of T-cell antigens in both malignant and non-malignant T cells. Key issues include T-cell aplasia, fratricide, and tumour contamination [96]. T-cell aplasia occurs when normal T cells are inadvertently destroyed by the infused CAR-T cells, leading to a heightened risk of severe infections. Fratricide refers to the destruction of CAR-T cells by other CAR-T cells, which compromises their persistence and expansion. Tumour contamination, on the other hand, involves the collection of malignant T cells during the manufacturing process, which are then inadvertently re-infused. Addressing these challenges is essential for optimising the clinical application of CAR-T therapies in T-cell malignancies, which primarily involves the careful selection of CAR-T targets, coupling up with various manufacturing strategies.

Several targets are currently being investigated, including CD5, CD7, CD30, CD70, and TRBC1. Many of these studies are in the early-phase clinical trial stage, focusing on dose optimisation and incorporating patients with T-cell acute lymphoblastic leukaemia (T-ALL) as part of the cohorts. Enrolment of patients with nodal PTCLs remains limited, resulting in a dearth of data specific to this subgroup. Among these targets, TRBC1 has garnered considerable attention due to its distinguishing feature of sparing mutually exclusive TRBC2 T cells, which helps mitigate the risks of severe T-cell aplasia and fratricide. Notably, the recent LibraT1 study has yielded promising preliminary results for AUTO4, reporting four CMRs out of ten evaluated, further advancing the potential of this approach [97]. The study is ongoing with an aim to identify optimal dosing to advance into a phase II study. Other strategies such as considering using NK cells or allogenic T cells are also underway.

## 5. Summary

Nodal T-cell lymphomas exhibit considerable diversity in their pathophysiology, making a uniform treatment approach challenging. Brentuximab vedotin has marked a significant advancement in the management of this disease group; however, its efficacy is limited, particularly in non-ALCL subtypes. The duration of response remains a critical consideration, as options for subsequent treatment in relapsed or refractory cases are scarce. Consolidation strategies, such as transplantation, are well supported in clinical practice, although the lack of randomised controlled trials limits the definitive evidence for its benefit. Ongoing research into various targeted agents and immunotherapies offers promising prospects, potentially enabling a more personalised treatment approach that acknowledges the unique characteristics of each T-cell lymphoma subtype.

## Figures and Tables

**Table 1:** Nodal peripheral T-cell lymphomas.

Subtype	Cell of Origin	Morphology	Immunophenotype	Genetic/Molecular Features	Targeted Therapies
Nodal T-follicular helper cell lymphoma (nTFHL) nTFHL, angioimmunoblastic type nTFHL, follicular type nTFHL, NOS	CD4+ TFH cell	Small to medium atypical neoplastic cells with clear cytoplasm Angioimmunoblastic type: Diffuse with follicular dendritic cell expansion, polymorphous infiltrate of inflammatory cells and vasculature proliferation Follicular type: Follicular or perifollicular growth pattern	CD4+ TFH cell markers: CD10, BCL6, PD1 (CD279), ICOS, CXCL13 CD20+ B immunoblasts (often EBV+) Angioimmunoblastic type: CD21+ follicular dendritic cells	Epigenetic mutations: TET2, DNMT3A and IDH2 R172 Mutations in genes of the TCR pathway: RHOAG17V, CD28, VAV1 Fusion transcripts: CD28-ICOS, CD28-CTLA4, ITK-SYK	Anti-PD1 monoclonal antibodies Epigenetic modifiers Hypomethylating agents Multikinase inhibitors SYK inhibitors CTLA4 inhibitors
PTCL, NOS	CD4+ T cell (most common) CD8+ T cell Th1 (TBX21) or Th2 (GATA3) subsets	Highly variable cytology of the neoplastic cells and variable microenvironment	Variable expression of pan-T cell antigens and of cytotoxic markers (TIA-1, granzyme B, perforin)	Epigenetic mutations: TET1, TET2, TET3 and DNMT3A NF-kB activation Gains/amplifications: STAT3 and MYC Loss or mutations in tumour suppressor genes: TP53, PTEN, CDKN2A/B Mutations in genes of the PI3K pathways TP63 rearrangements	Anti-CCR4 antibodies Epigenetic modifiers Hypomethylating agents PI3K inhibitors JAK/STAT inhibitors
Anaplastic large cell lymphoma, ALK positive (ALK+ ALCL)	CD4+ αβ T cell	Large pleomorphic cells, cohesive sheets with an intrasinusoidal growth pattern	CD30+ ALK+ Often extensive loss of pan-T-cell antigens Frequently expression of cytotoxic markers EBV-	Fusion transcripts involving ALK: NPM-ALK most common Downstream signalling pathways JAK-STAT3, RAS-ERK, and PI3K-AKT Mutations in genes of the NOTCH1 pathway	Brentuximab-Vedotin ALK inhibitors JAK/STAT inhibitors
Anaplastic large cell lymphoma, ALK negative (ALK− ALCL)	CD4+ αβ T cell	Large pleomorphic cells, cohesive sheets with an intrasinusoidal growth pattern	CD30+ ALK- Variable expression of pan-T-cell antigens and of cytotoxic markers EBV−	Activating mutations: STAT3 and JAK1 Other fusion transcripts leading to STAT3 activation: NFKB2-ROS2 and NFKB2-TYK2 Fusion transcripts involving DUSP22: DUSP22-IRF4 TP63 rearrangements Abnormal ERBB4 transcripts	Brentuximab-Vedotin JAK/STAT inhibitors Kinase inhibitor
EBV-positive nodal T- and NK-cell lymphoma	CD8+ T cell NK cell	Diffuse infiltrate of medium to large monomorphic cells, lack of angiocentric growth	EBV+ CD8+, CD56− Cytotoxic markers	Mutations in TET2, DNMT3A, PIK3CD, DDX3X, and STAT3	Epigenetic modifiers

**Table 2:** Novel therapeutic agents in the treatment of nodal peripheral T-cell lymphomas.

Mechanism of Action	Drug	Responses	mPFS/mOS	nTFH/ AITL	PTCL-NOS	ALCL	Other Subtypes	Relevant/Ongoing Studies
Histone deacetylase inhibitor (HDACi)	Romidepsin [50]	*N* = 131 ORR 25% CR 15%; PR 11%	PFS: 4 m	ORR 30% CR 19%	ORR 29% CR 14%	ALK- ORR 24% CR 19%	Others ORR 0% CR 0%	
	Belinostat [51]	*N* = 129 ORR 26% CR 11%; PR 15%	PFS: 1.6 m OS: 7.9 m	ORR 46%	ORR 23%	ALK- ORR 15% ALK+ ORR 0%	Enteropathy ORR 0% ENKTL ORR 50% Hepatosplenic ORR 0%	
	Chidamide [52]	*N* = 79 ORR 28% CR 14%; PR 14%	PFS: 2.1 m OS: 21.4 m	ORR 50% CR 40%	ORR 22% CR 7%	ALK+ or unknown ORR 33% CR 0% ALK- ORR 45% CR 36%	ENKTL ORR 19% CR 6% Others ORR 11% CR 0%	
Hypomethylating agent	Oral Azacitidine [53]	*N* = 42 ORR 33% CR 12%	PFS: 5.6 m OS: 18.4 m	ORR 33% CR 12%	-	-	-	
EZH inhibitor	Valemetostat [54]	*N* = 119 ORR 44% CR 14%; PR 29%	PFS: 5.5 m OS: 17 m	ORR 54% CR 18%	ORR 32% CR 10%	ORR 33% CR 11%	Others ORR 47% CR 16%	
PI3-kinase pathway	Duvelisib [55]	*N* = 123 ORR 48% CR 33%	PFS: 3.5 m OS: 12.4 m	ORR 62% CR 53%	ORR 49% CR 27%	ORR 15% CR 13%	-	Phase III NCT06522737
	Linperlisib [56]	*N* = 43 ORR 61% CR 35%; PR 26%	PFS: 11.8 m OS: NE	ORR 81% CR 50%	ORR 41% CR 24%	ORR 50% CR 33%	Others ORR 75% CR 25%	Phase II NCT05274997
Folate DHFR inhibitor	Pralatrexate [57]	*N* = 109 ORR 29% CR 11%; PR 18%	PFS 3.5 m OS: 14.5 m	ORR 8%	ORR 32%	ORR 35%	MF ORR 25% Others ORR 38%	
JAK/STAT pathway	Ruxolitinib [58]	*N* = 52 ORR 25% CR 6%; PR 19%	PFS: 2.8 m OS: 26.2 m	ORR 33% CR 11%	ORR 18% CR 9%	ORR 25% CR 25%	MF ORR 14% CR 0%	
	Golidocritinib [59]	*N* = 88 ORR 44% CR 24%; PR 20%	PFS: 5.6 m OS: 19.4 m	ORR 56%	ORR 46%	ORR 10%	NKTCL 67% Others 44%	Phase II NCT06511869
CelMod	Lenalidomide [60,61]	*N* = 40 + 54 ORR 22–26% CR 8–11%; PR 18%	PFS: 2.5–4 m OS: 12 m	ORR 33% CR 11%	ORR 43% CR 14%	ORR 10% CR 0%	Enteropathy ORR 0% Hepatosplenic ORR 0%	Phase II NCT03730740
Anti-PD1	Pembrolizumab [62]	*N* = 17 ORR 33% CR 27%	PFS: 3.2 m OS: 10.6 m	-	-	-	-	Phase II NCT02362997
	Durvalumab [63]	*N* = 12 ORR 42%	PFS: 6.2 m	-	-	-	-	

**Table 3:** Combination therapy approaches in the treatment of nodal peripheral T-cell lymphomas.

Combination Treatment	Core Findings	Subtype Analysis
Romidepsin + Duvelisib [82,83]	ORR 56–59%; CR 41–44% (R/R) PFS: 8.5 m; OS: 12 m	ORR 70%; CR 40–60% (nTFH) ORR 40–50%; CR 28–40% (PTCL-NOS)
Romidepsin + Lenalidomide [84]	ORR 65%; CR 26% (frontline) PFS: 4.8 m; OS: 18.3 m	ORR 79%; CR 34% (AITL) ORR 50%; CR 17% (PTCL-NOS)
Romidepsin + Pralatrexate [85]	ORR 71%; CR 29% (R/R) PFS: 4.4 m; OS: 12.4 m	Not available
Romidepsin + Pembrolizumab [86]	ORR 47.3%; CR 37.3% (R/R) PFS: 3.6 m; OS: 21.3 m	ORR 85.7%; CR 57.1% (nTFH)
Romidepsin + Azacitidine [87]	ORR 70%; CR 50% (frontline) PFS: NR; OS: NR ORR 54%; CR 38% (R/R) PFS: 8 m; OS: 20.6 m	ORR 80%; CR 60% (nTFH) ORR 25%; CR 13% (others)
Romidepsin + Azacitidine, Dexamethasone, Lenalidomide [88]	ORR 63%; CR 19% (R/R)	Not available
Duvelisib + Azacitidine [89]	ORR 46%; CR 31% (R/R) PFS: 2.2 m: OS 10.2 m	ORR 100%; CR 100% (nTFH)
Duvelisib + Ruxolitinib [90]	ORR 41%; CR 24% (R/R)	ORR 52%; CR 29% (Presence of JAK/STAT activation) ORR 14%; CR 14% (Absence of JAK/STAT activation)
Lenalidomide + Brentuximab [91]	ORR 50%; CR 38% (R/R)	Not available
Chidamide + Brentuximab + chemotherapy [92]	ORR 88%, CR 76% (frontline) ORR 36%, CR 13% (R/R)	ORR 100% (frontline ALCL) ORR 86% (frontline AITL) ORR 80% (frontline PTCL-NOS)
Chidamide + Azacitidine + chemotherapy [93]	ORR 97%; CR 69% (frontline) PFS: NR; OS: NR	Not available
**Ongoing studies**		
Chidamide + Duvelisib	NCT05976997	
Chidamide + Azacitidine	NCT04480125	
Pralatrexate + Decitabine + Pembrolizumab	NCT03240211	
Durvalumab + Pralatrexate, Azacitidine or Romidepsin	NCT03161223	

## Data Availability

Not applicable.

## References

[1] International T-Cell Lymphoma Project (2008). International Peripheral T-Cell and Natural Killer/T-Cell Lymphoma Study: Pathology Findings and Clinical Outcomes. J. Clin. Oncol..

[2] Federico M., Rudiger T., Bellei M., Nathwani B.N., Luminari S., Coiffier B., Harris N.L., Jaffe E.S., Pileri S.A., Savage K.J. (2013). Clinicopathologic Characteristics of Angioimmunoblastic T-Cell Lymphoma: Analysis of the International Peripheral T-Cell Lymphoma Project. J. Clin. Oncol..

[3] Savage K.J., Harris N.L., Vose J.M., Ullrich F., Jaffe E.S., Connors J.M., Rimsza L., Pileri S.A., Chhanabhai M., Gascoyne R.D. (2008). ALK−Anaplastic Large-Cell Lymphoma is Clinically and Immunophenotypically Different from Both ALK + ALCL and Peripheral T-Cell Lymphoma, Not Otherwise Specified: Report from the International Peripheral T-Cell Lymphoma Project. Blood.

[4] Alaggio R., Amador C., Anagnostopoulos I., Attygalle A.D., Araujo I.B.d.O., Berti E., Bhagat G., Borges A.M., Boyer D., Calaminici M. (2022). The 5th Edition of the World Health Organization Classification of Haematolymphoid Tumours: Lymphoid Neoplasms. Leukemia.

[5] Campo E., Jaffe E.S., Cook J.R., Quintanilla-Martinez L., Swerdlow S.H., Anderson K.C., Brousset P., Cerroni L., de Leval L., Dirnhofer S. (2022). The International Consensus Classification of Mature Lymphoid Neoplasms: A Report from the Clinical Advisory Committee. Blood.

[6] Lage L.A.d.P.C., Cabral T.C.d.S., Costa R.d.O., Gonçalves M.d.C., Levy D., Zerbini M.C.N., Pereira J. (2015). Primary Nodal Peripheral T-Cell Lymphomas: Diagnosis and Therapeutic Considerations. Rev. Bras. Hematol. Hemoter..

[7] Hsi E.D., Horwitz S.M., Carson K.R., Pinter-Brown L.C., Rosen S.T., Pro B., Federico M., Gisselbrecht C., Schwartz M., Bellm L.A. (2017). Analysis of Peripheral T-Cell Lymphoma Diagnostic Workup in the United States. Clin. Lymphoma Myeloma Leuk..

[8] de Leval L., Gaulard P., Dogan A. (2024). A Practical Approach to the Modern Diagnosis and Classification of T-and NK-Cell Lymphomas. Blood.

[9] Ellin F., Landström J., Jerkeman M., Relander T. (2014). Real-World Data on Prognostic Factors and Treatment in Peripheral T-Cell Lymphomas: A Study from the Swedish Lymphoma Registry. Blood.

[10] Weiss J., Reneau J., Wilcox R.A. (2023). PTCL, NOS: An Update on Classification, Risk-Stratification, and Treatment. Front. Oncol..

[11] Gutiérrez-García G., García-Herrera A., Cardesa T., Martínez A., Villamor N., Ghita G., Martínez-Trillos A., Colomo L., Setoain X., Rodríguez S. (2011). Comparison of Four Prognostic Scores in Peripheral T-Cell Lymphoma. Ann. Oncol..

[12] Chang E.W.Y., Tan Y.H., Chan J.Y. (2024). Novel Clinical Risk Stratification and Treatment Strategies in Relapsed/Refractory Peripheral T-Cell Lymphoma. J. Hematol. Oncol..

[13] Sibon D., Fournier M., Brière J., Lamant L., Haioun C., Coiffier B., Bologna S., Morel P., Gabarre J., Hermine O. (2012). Long-Term Outcome of Adults with Systemic Anaplastic Large-Cell Lymphoma Treated Within the Groupe d’Étude Des Lymphomes de l’Adulte Trials. J. Clin. Oncol..

[14] Chiba S., Sakata-Yanagimoto M. (2020). Advances in Understanding of Angioimmunoblastic T-Cell Lymphoma. Leukemia.

[15] Lewis N.E., Petrova-Drus K., Huet S., Epstein-Peterson Z.D., Gao Q., Sigler A.E., Baik J., Ozkaya N., Moskowitz A.J., Kumar A. (2020). Clonal Hematopoiesis in Angioimmunoblastic T-Cell Lymphoma with Divergent Evolution to Myeloid Neoplasms. Blood Adv..

[16] Hathuc V., Kreisel F. (2022). Genetic Landscape of Peripheral T-Cell Lymphoma. Life.

[17] Hapgood G., Savage K.J. (2015). The Biology and Management of Systemic Anaplastic Large Cell Lymphoma. Blood.

[18] Sabattini E., Pizzi M., Tabanelli V., Baldin P., Sacchetti C.S., Agostinelli C., Zinzani P.L., Pileri S.A. (2013). CD30 Expression in Peripheral T-Cell Lymphomas. Haematologica.

[19] Feldman A.L., Dogan A., Smith D.I., Law M.E., Ansell S.M., Johnson S.H., Porcher J.C., Ozsan N., Wieben E.D., Eckloff B.W. (2011). Discovery of Recurrent t(6;7)(P25.3;Q32.3) Translocations in ALK-Negative Anaplastic Large Cell Lymphomas by Massively Parallel Genomic Sequencing. Blood.

[20] Parrilla Castellar E.R., Jaffe E.S., Said J.W., Swerdlow S.H., Ketterling R.P., Knudson R.A., Sidhu J.S., Hsi E.D., Karikehalli S., Jiang L. (2014). ALK-Negative Anaplastic Large Cell Lymphoma Is a Genetically Heterogeneous Disease with Widely Disparate Clinical Outcomes. Blood.

[21] Qiu L., Tang G., Li S., Vega F., Lin P., Wang S.A., Wang W., Iyer S.P., Malpica L., Miranda R.N. (2023). DUSP22 Rearrangement is Associated with a Distinctive Immunophenotype but Not Outcome in Patients with Systemic ALK-Negative Anaplastic Large Cell Lymphoma. Haematologica.

[22] Hapgood G., Ben-Neriah S., Mottok A., Lee D.G., Robert K., Villa D., Sehn L.H., Connors J.M., Gascoyne R.D., Feldman A.L. (2019). Identification of High-Risk DUSP22-Rearranged ALK-Negative Anaplastic Large Cell Lymphoma. Br. J. Haematol..

[23] Sibon D., Bisig B., Bonnet C., Poullot E., Bachy E., Cavalieri D., Fataccioli V., Bregnard C., Drieux F., Bruneau J. (2023). ALK-Negative Anaplastic Large Cell Lymphoma with DUSP22 Rearrangement Has Distinctive Disease Characteristics with Better Progression-Free Survival: A LYSA Study. Haematologica.

[24] Bigas A., Rodriguez-Sevilla J.J., Espinosa L., Gallardo F. (2022). Recent Advances in T-Cell Lymphoid Neoplasms. Exp. Hematol..

[25] Bisig B., Savage K.J., de Leval L. (2023). Pathobiology of Nodal Peripheral T-Cell Lymphomas: Current Understanding and Future Directions. Haematologica.

[26] Fisher R.I., Gaynor E.R., Dahlberg S., Oken M.M., Grogan T.M., Mize E.M., Glick J.H., Coltman C.A., Miller T.P. (1993). Comparison of a Standard Regimen (CHOP) with Three Intensive Chemotherapy Regimens for Advanced Non-Hodgkin’s Lymphoma. N. Engl. J. Med..

[27] Reimer P., Rüdiger T., Geissinger E., Weissinger F., Nerl C., Schmitz N., Engert A., Einsele H., Müller-Hermelink H.K., Wilhelm M. (2009). Autologous Stem-Cell Transplantation As First-Line Therapy in Peripheral T-Cell Lymphomas: Results of a Prospective Multicenter Study. J. Clin. Oncol..

[28] Gleeson M., Peckitt C., To Y.M., Edwards L., Oates J., Wotherspoon A., Attygalle A.D., Zerizer I., Sharma B., Chua S. (2018). CHOP versus GEM-P in Previously Untreated Patients with Peripheral T-Cell Lymphoma (CHEMO-T): A Phase 2, Multicentre, Randomised, Open-Label Trial. Lancet Haematol..

[29] Mahadevan D., Unger J.M., Spier C.M., Persky D.O., Young F., Leblanc M., Fisher R.I., Miller T.P. (2013). Phase 2 Trial of Combined Cisplatin, Etoposide, Gemcitabine, and Methylprednisolone (PEGS) in Peripheral T-Cell Non-Hodgkin Lymphoma: Southwest Oncology Group Study S0350. Cancer.

[30] Simon A., Peoch M., Casassus P., Deconinck E., Colombat P., Desablens B., Tournilhac O., Eghbali H., Foussard C., Jaubert J. (2010). Upfront VIP-Reinforced-ABVD (VIP-RABVD) is Not Superior to CHOP/21 in Newly Diagnosed Peripheral T Cell Lymphoma. Results of the Randomized Phase III Trial GOELAMS-LTP95. Br. J. Haematol..

[31] Schmitz N., Trümper L., Ziepert M., Nickelsen M., Ho A.D., Metzner B., Peter N., Loeffler M., Rosenwald A., Pfreundschuh M. (2010). Treatment and Prognosis of Mature T-Cell and NK-Cell Lymphoma: An Analysis of Patients with T-Cell Lymphoma Treated in Studies of the German High-Grade Non-Hodgkin Lymphoma Study Group. Blood.

[32] d’Amore F., Leppä S., Silva M.G.d., Relander T., Lauritzsen G.F., Brown P.D.N., Pezzutto A., Doorduijn J.K., Weidmann E., van Gelder M. (2018). Final Analysis of the Front-Line Phase III Randomized ACT-1 Trial in Younger Patients with Systemic Peripheral T-Cell Lymphoma Treated with CHOP Chemotherapy with or without Alemtuzumab and Consolidated by Autologous Hematopoietic Stem Cell Transplant. Blood.

[33] Wulf G.G., Altmann B., Ziepert M., D’Amore F., Held G., Greil R., Tournilhac O., Relander T., Viardot A., Wilhelm M. (2021). Alemtuzumab plus CHOP versus CHOP in Elderly Patients with Peripheral T-Cell Lymphoma: The DSHNHL2006-1B/ACT-2 Trial. Leukemia.

[34] Ganjoo K., Hong F., Horning S.J., Gascoyne R.D., Natkunam Y., Swinnen L.J., Habermann T.M., Kahl B.S., Advani R.H. (2014). Bevacizumab and Cyclosphosphamide, Doxorubicin, Vincristine and Prednisone in Combination for Patients with Peripheral T-Cell or Natural Killer Cell Neoplasms: An Eastern Cooperative Oncology Group Study (E2404). Leuk. Lymphoma.

[35] Lemonnier F., Safar V., Beldi-Ferchiou A., Cottereau A.S., Bachy E., Cartron G., Fataccioli V., Pelletier L., Robe C., Letourneau A. (2021). Integrative Analysis of a Phase 2 Trial Combining Lenalidomide with CHOP in Angioimmunoblastic T-Cell Lymphoma. Blood Adv..

[36] Bachy E., Camus V., Thieblemont C., Sibon D., Casasnovas R.-O., Ysebaert L., Damaj G., Guidez S., Pica G.M., Kim W.S. (2021). Romidepsin Plus CHOP Versus CHOP in Patients with Previously Untreated Peripheral T-Cell Lymphoma: Results of the Ro-CHOP Phase III Study (Conducted by LYSA). J. Clin. Oncol..

[37] Pro B., Advani R., Brice P., Bartlett N.L., Rosenblatt J.D., Illidge T., Matous J., Ramchandren R., Fanale M., Connors J.M. (2012). Brentuximab Vedotin (SGN-35) in Patients with Relapsed or Refractory Systemic Anaplastic Large-Cell Lymphoma: Results of a Phase II Study. J. Clin. Oncol..

[38] Pro B., Advani R., Brice P., Bartlett N.L., Rosenblatt J.D., Illidge T., Matous J., Ramchandren R., Fanale M., Connors J.M. (2017). Five-Year Results of Brentuximab Vedotin in Patients with Relapsed or Refractory Systemic Anaplastic Large Cell Lymphoma. Blood.

[39] Horwitz S., O’Connor O.A., Pro B., Illidge T., Fanale M., Advani R., Bartlett N.L., Christensen J.H., Morschhauser F., Domingo-Domenech E. (2019). Brentuximab Vedotin with Chemotherapy for CD30-Positive Peripheral T-Cell Lymphoma (ECHELON-2): A Global, Double-Blind, Randomised, Phase 3 Trial. Lancet.

[40] Horwitz S., O’Connor O.A., Pro B., Trümper L., Iyer S., Advani R., Bartlett N.L., Christensen J.H., Morschhauser F., Domingo-Domenech E. (2022). The ECHELON-2 Trial: 5-Year Results of a Randomized, Phase III Study of Brentuximab Vedotin with Chemotherapy for CD30-Positive Peripheral T-Cell Lymphoma. Ann. Oncol..

[41] Herrera A.F., Zain J., Savage K.J., Feldman T., Brammer J.E., Chen L., Puverel S., Popplewell L., Budde L.E., Mei M. (2024). Brentuximab Vedotin plus Cyclophosphamide, Doxorubicin, Etoposide, and Prednisone Followed by Brentuximab Vedotin Consolidation in CD30-Positive Peripheral T-Cell Lymphomas: A Multicentre, Single-Arm, Phase 2 Study. Lancet Haematol..

[42] Iyer S.P., Jagadeesh D., Domingo-Domenech E., Benedetti F., Rodriguez Izquierdo A., Bouabdallah K., Vitolo U., Illidge T.M., Liu J., Shaikh E. (2024). Frontline Brentuximab Vedotin and CHP (A + CHP) in Patients (Pts) with Peripheral T-Cell Lymphoma with Less than 10% CD30 Expression: Results from the Phase 2 SGN35-032 Study. J. Clin. Oncol..

[43] Savage K.J., Horwitz M.S., Advani R., Christensen J.H., Domingo-Domenech E., Rossi G., Morschhauser F., Alpdogan O., Suh C., Tobinai K. (2022). Role of Stem Cell Transplant in CD30-Positive PTCL Following Frontline Brentuximab Vedotin+ CHP or CHOP in ECHELON-2. Blood Adv..

[44] D’Amore F., Relander T., Lauritzsen G.F., Jantunen E., Hagberg H., Anderson H., Holte H., Österborg A., Merup M., Brown P. (2012). Up-Front Autologous Stem-Cell Transplantation in Peripheral T-Cell Lymphoma: NLG-T-01. J. Clin. Oncol..

[45] Wilhelm M., Smetak M., Reimer P., Geissinger E., Ruediger T., Metzner B., Schmitz N., Engert A., Schaefer-Eckart K., Birkmann J. (2016). First-Line Therapy of Peripheral T-Cell Lymphoma: Extension and Long-Term Follow-up of a Study Investigating the Role of Autologous Stem Cell Transplantation. Blood Cancer J..

[46] Fossard G., Broussais F., Coelho I., Bailly S., Nicolas-Virelizier E., Toussaint E., Lancesseur C., Le Bras F., Willems E., Tchernonog E. (2018). Role of Up-Front Autologous Stem-Cell Transplantation in Peripheral T-Cell Lymphoma for Patients in Response after Induction: An Analysis of Patients from LYSA Centers. Ann. Oncol..

[47] Park S.I., Horwitz S.M., Foss F.M., Pinter-Brown L.C., Carson K.R., Rosen S.T., Pro B., Hsi E.D., Federico M., Gisselbrecht C. (2019). The Role of Autologous Stem Cell Transplantation in Patients with Nodal Peripheral T-cell Lymphomas in First Complete Remission: Report from COMPLETE, a Prospective, Multicenter Cohort Study. Cancer.

[48] García-Sancho A.M., Bellei M., López-Parra M., Gritti G., Cortés M., Novelli S., Panizo C., Petrucci L., Gutiérrez A., Dlouhy I. (2022). Autologous Stem-Cell Transplantation as Consolidation of First-Line Chemotherapy in Patients with Peripheral T-Cell Lymphoma: A Multicenter GELTAMO/FIL Study. Haematologica.

[49] Schmitz N., Truemper L.H., Bouabdallah K., Ziepert M., Leclerc M., Cartron G., Jaccard A., Reimer P., Wagner-Drouet E.M., Wilhelm M. (2021). A Randomized Phase 3 Trial of Auto vs. Allo Transplantation as Part of First-Line Therapy in Poor-Risk Peripheral T-NHL. Blood.

[50] Coiffier B., Pro B., Prince H.M., Foss F., Sokol L., Greenwood M., Caballero D., Borchmann P., Morschhauser F., Wilhelm M. (2012). Results from a Pivotal, Open-Label, Phase II Study of Romidepsin in Relapsed or Refractory Peripheral T-Cell Lymphoma after Prior Systemic Therapy. J. Clin. Oncol..

[51] O’Connor O.A., Horwitz S., Masszi T., Van Hoof A., Brown P., Doorduijn J., Hess G., Jurczak W., Knoblauch P., Chawla S. (2015). Belinostat in Patients with Relapsed or Refractory Peripheral T-Cell Lymphoma: Results of the Pivotal Phase II BELIEF (CLN-19) Study. J. Clin. Oncol..

[52] Shi Y., Dong M., Hong X., Zhang W., Feng J., Zhu J., Yu L., Ke X., Huang H., Shen Z. (2015). Results from a Multicenter, Open-Label, Pivotal Phase II Study of Chidamide in Relapsed or Refractory Peripheral T-Cell Lymphoma. Ann. Oncol..

[53] Dupuis J., Tsukasaki K., Bachy E., Morschhauser F., Cartron G., Fukuhara N., Daguindau N., Casasnovas R.-O., Snauwaert S., Gressin R. (2022). Oral Azacytidine in Patients with Relapsed/Refractory Angioimmunoblastic T-Cell Lymphoma: Final Analysis of the Oracle Phase III Study. Blood.

[54] Horwitz S.M., Izutsu K., Mehta-Shah N., Cordoba R., Barta S.K., Bachy E., Gritti G., Jacobsen E., Kusumoto S., Guillermin Y. (2023). Efficacy and Safety of Valemetostat Monotherapy in Patients with Relapsed or Refractory Peripheral T-Cell Lymphomas: Primary Results of the Phase 2 VALENTINE-PTCL01 Study. Blood.

[55] Mehta-Shah N., Zinzani P.L., Jacobsen E.D., Zain J., Mead M., Casulo C., Gritti G., Pinter-Brown L.C., Izutsu K., Bentur O.S. (2024). Duvelisib in Patients with Relapsed/Refractory Peripheral T-Cell Lymphoma: Final Results from the Phase 2 PRIMO Trial. Blood.

[56] Jin J., Cen H., Zhou K., Xu X., Li F., Wu T., Yang H., Wang Z., Li Z., Huang W. (2024). A Phase Ib Study of Linperlisib in the Treatment of Patients with Relapsed and/or Refractory Peripheral T-Cell Lymphoma. Clin. Cancer Res..

[57] O’Connor O.A., Pro B., Pinter-Brown L., Bartlett N., Popplewell L., Coiffier B., Jo Lechowicz M., Savage K.J., Shustov A.R., Gisselbrecht C. (2011). Pralatrexate in Patients with Relapsed or Refractory Peripheral T-Cell Lymphoma: Results from the Pivotal PROPEL Study. J. Clin. Oncol..

[58] Moskowitz A.J., Ghione P., Jacobsen E., Ruan J., Schatz J.H., Noor S., Myskowski P., Vardhana S., Ganesan N., Hancock H. (2021). A Phase 2 Biomarker-Driven Study of Ruxolitinib Demonstrates Effectiveness of JAK/STAT Targeting in T-Cell Lymphomas. Blood.

[59] Song Y., Malpica L., Cai Q., Zhao W., Zhou K., Wu J., Zhang H., Mehta-Shah N., Ding K., Liu Y. (2024). Golidocitinib, a Selective JAK1 Tyrosine-Kinase Inhibitor, in Patients with Refractory or Relapsed Peripheral T-Cell Lymphoma (JACKPOT8 Part B): A Single-Arm, Multinational, Phase 2 Study. Lancet Oncol..

[60] Morschhauser F., Fitoussi O., Haioun C., Thieblemont C., Quach H., Delarue R., Glaisner S., Gabarre J., Bosly A., Lister J. (2013). A Phase 2, Multicentre, Single-Arm, Open-Label Study to Evaluate the Safety and Efficacy of Single-Agent Lenalidomide (Revlimid^®^) in Subjects with Relapsed or Refractory Peripheral T-Cell Non-Hodgkin Lymphoma: The EXPECT Trial. Eur. J. Cancer.

[61] Toumishey E., Prasad A., Dueck G., Chua N., Finch D., Johnston J., Van Der Jagt R., Stewart D., White D., Belch A. (2015). Final Report of a Phase 2 Clinical Trial of Lenalidomide Monotherapy for Patients with T-Cell Lymphoma. Cancer.

[62] Barta S.K., Zain J., MacFarlane A.W., Smith S.M., Ruan J., Fung H.C., Tan C.R., Yang Y., Alpaugh R.K., Dulaimi E. (2019). Phase II Study of the PD-1 Inhibitor Pembrolizumab for the Treatment of Relapsed or Refractory Mature T-Cell Lymphoma. Clin. Lymphoma Myeloma Leuk..

[63] Querfeld C., Chen L., Wu X., Han Z., Su C., Banez M., Quach J., Barnhizer T., Crisan L., Rosen S.T. (2024). Randomized Phase 2 Trial of the Anti-PD-L1 Monoclonal Antibody Durvalumab Plus Lenalidomide Versus Single-Agent Durvalumab in Patients with Refractory/Advanced Cutaneous T Cell Lymphoma. Blood.

[64] Johnston P.B., Cashen A.F., Nikolinakos P.G., Beaven A.W., Barta S.K., Bhat G., Hasal S.J., De Vos S., Oki Y., Deng C. (2021). Belinostat in Combination with Standard Cyclophosphamide, Doxorubicin, Vincristine and Prednisone as First-Line Treatment for Patients with Newly Diagnosed Peripheral T-Cell Lymphoma. Exp. Hematol. Oncol..

[65] Ghione P., Faruque P., Mehta-Shah N., Seshan V., Ozkaya N., Bhaskar S., Yeung J., Spinner M.A., Lunning M., Inghirami G. (2020). T Follicular Helper Phenotype Predicts Response to Histone Deacetylase Inhibitors in Relapsed/Refractory Peripheral T-Cell Lymphoma. Blood Adv..

[66] Iyer S.P., Johnston P.B., Barta S.K. (2024). Pralatrexate Injection Combined with CHOP for Treatment of PTCL: Results from the Fol-CHOP Dose-Finding Phase 1 Trial. Blood Adv..

[67] Advani R.H., Ansell S.M., Lechowicz M.J., Beaven A.W., Loberiza F., Carson K.R., Evens A.M., Foss F., Horwitz S., Pro B. (2016). A Phase II Study of Cyclophosphamide, Etoposide, Vincristine and Prednisone (CEOP) Alternating with Pralatrexate (P) as Front Line Therapy for Patients with Peripheral T-Cell Lymphoma (PTCL): Final Results from the T-Cell Consortium Trial. Br. J. Haematol..

[68] Yoon S.E., Cho J., Kim Y.J., Kim S.J., Kim W.S. (2022). Real-World Efficacy of 5-Azacytidine as Salvage Chemotherapy for Angioimmunoblastic T-Cell Lymphoma. Clin. Lymphoma Myeloma Leuk..

[69] Dupuis J., Bachy E., Morschhauser F., Cartron G., Fukuhara N., Daguindau N., Casasnovas R.-O., Snauwaert S., Gressin R., Fox C.P. (2024). Oral Azacitidine Compared with Standard Therapy in Patients with Relapsed or Refractory Follicular Helper T-Cell Lymphoma (ORACLE): An Open-Label Randomised, Phase 3 Study. Lancet Haematol..

[70] Ruan J., Moskowitz A.J., Mehta-Shah N., Sokol L., Chen Z., Rahim R., Song W., Van Besien K., Horwitz S.M., Rutherford S.C. (2020). Multi-Center Phase II Study of Oral Azacitidine (CC-486) Plus CHOP as Initial Treatment for Peripheral T-Cell Lymphoma (PTCL). Blood.

[71] Ruan J., Orlando E.H., Moskowitz A., Chen Z., Mehta-Shah N., Sokol L., Horwitz S., Rutherford S.C., Sahni T., Melnick A.M. (2024). Subsequent Treatment and Clinical Outcome Following Induction Therapy on a Phase II Study of Oral Azacitidine Plus CHOP for Peripheral T-Cell Lymphoma (PTCL). Blood.

[72] Foster J.H., Voss S.D., Hall D.C., Minard C.G., Balis F.M., Wilner K., Berg S.L., Fox E., Adamson P.C., Blaney S.M. (2021). Activity of Crizotinib in Patients with ALK-Aberrant Relapsed/Refractory Neuroblastoma: A Children’s Oncology Group Study (ADVL0912). Clin. Cancer Res..

[73] Lowe E.J., Reilly A.F., Lim M.S., Gross T.G., Saguilig L., Barkauskas D.A., Wu R., Alexander S., Bollard C.M. (2022). Crizotinib in Combination with Chemotherapy for Pediatric Patients with ALK1 Anaplastic Large-Cell Lymphoma: The Results of Children’s Oncology Group Trial ANHL12P1. J. Clin. Oncol..

[74] Camidge D.R., Kim H.R., Ahn M.-J., Yang J.C.H., Han J.-Y., Hochmair M.J., Lee K.H., Delmonte A., Garcia Campelo M.R., Kim D.-W. (2021). Brigatinib Versus Crizotinib in ALK Inhibitor-Naive Advanced ALK-Positive NSCLC: Final Results of Phase 3 ALTA-1L Trial. J. Thorac. Oncol..

[75] Shaw A.T., Kim T.M., Crinò L., Gridelli C., Kiura K., Liu G., Novello S., Bearz A., Gautschi O., Mok T. (2017). Ceritinib versus Chemotherapy in Patients with ALK-Rearranged Non-Small-Cell Lung Cancer Previously given Chemotherapy and Crizotinib (ASCEND-5): A Randomised, Controlled, Open-Label, Phase 3 Trial. Lancet Oncol..

[76] Peters S., Camidge D.R., Shaw A.T., Gadgeel S., Ahn J.S., Kim D.-W., Ou S.-H.I., Pérol M., Dziadziuszko R., Rosell R. (2017). Alectinib versus Crizotinib in Untreated *ALK*-Positive Non–Small-Cell Lung Cancer. N. Engl. J. Med..

[77] Solomon B.J., Liu G., Felip E., Mok T.S.K., Soo R.A., Mazieres J., Shaw A.T., de Marinis F., Goto Y., Wu Y.-L. (2024). Lorlatinib Versus Crizotinib in Patients with Advanced *ALK*-Positive Non–Small Cell Lung Cancer: 5-Year Outcomes from the Phase III CROWN Study. J. Clin. Oncol..

[78] Fukano R., Mori T., Sekimizu M., Choi I., Kada A., Saito A.M., Asada R., Takeuchi K., Terauchi T., Tateishi U. (2020). Alectinib for Relapsed or Refractory Anaplastic Lymphoma Kinase-Positive Anaplastic Large Cell Lymphoma: An Open-Label Phase II Trial. Cancer Sci..

[79] Richly H., Kim T.M., Schuler M., Kim D.-W., Harrison S.J., Shaw A.T., Boral A.L., Yovine A., Solomon B. (2015). Ceritinib in Patients with Advanced Anaplastic Lymphoma Kinase–Rearranged Anaplastic Large-Cell Lymphoma. Blood.

[80] Brammer J.E., Zinzani P.L., Zain J., Mead M., Casulo C., Jacobsen E.D., Gritti G., Litwak D., Cohan D., Katz D.J. (2021). Duvelisib in Patients with Relapsed/Refractory Peripheral T-Cell Lymphoma from the Phase 2 Primo Trial: Results of an Interim Analysis. Blood.

[81] Mehta-Shah N., Geyer S.M., Barta S.K., Amengual J.E., Dockter T., Wright C., Dinner S., Hsi E.D., Bartlett N.L., Horwitz S.M. (2022). Alliance A059102: A Randomized Phase II U.S. Intergroup Study of CHO(E)P versus CC-486-CHO(E)P versus Duvelisib-CHO(E)P in Previously Untreated, CD30-Negative, Peripheral T-Cell Lymphomas. J. Clin. Oncol..

[82] Horwitz S.M., Moskowitz A.J., Jacobsen E.D., Mehta-Shah N., Khodadoust M.S., Fisher D.C., Myskowski P., Wang E.B.K., Tawa M., Davey T. (2018). The Combination of Duvelisib, a PI3K-δ,γ Inhibitor, and Romidepsin is Highly Active in Relapsed/Refractory Peripheral T-Cell Lymphoma with Low Rates of Transaminitis: Results of Parallel Multicenter, Phase 1 Combination Studies with Expansion Cohorts. Blood.

[83] Ford J.G., McCabe S.M., Lenart A.W., Sorial M.N., Merrill M.H., Rider A.B., Sohani A.R., Nemec R.A., Chopra K., MacVicar C. (2024). Real-World Evidence for Duvelisib and Romidepsin Combination in Patients with Relapsed/Refractory Peripheral T-Cell Lymphomas. Blood.

[84] Ruan J., Zain J., Palmer B., Jovanovic B., Mi X., Swaroop A., Winter J.N., Gordon L.I., Karmali R., Moreira J. (2023). Multicenter Phase 2 Study of Romidepsin plus Lenalidomide for Previously Untreated Peripheral T-Cell Lymphoma. Blood Adv..

[85] Amengual J.E., Lichtenstein R., Lue J., Sawas A., Deng C., Lichtenstein E., Khan K., Atkins L., Rada A., Kim H.A. (2018). A Phase 1 Study of Romidepsin and Pralatrexate Reveals Marked Activity in Relapsed and Refractory T-Cell Lymphoma. Blood.

[86] Iyer S., Prakash R., Feng L., Agbedia O., Xu J., Lee H.J., Malpica L., Ahmed S., Oki Y., Chihara D. (2023). TCL-275 Updated Results of an Investigator-Initiated Phase II Study of Pembrolizumab and Romidepsin for Patients with Relapsed or Refractory T-Cell Lymphoma (TCL) with Survival Analysis. Clin. Lymphoma Myeloma Leuk..

[87] Falchi L., Ma H., Klein S., Lue J.K., Montanari F., Marchi E., Deng C., Kim H.A., Rada A., Jacob A.T. (2021). Combined Oral 5-Azacytidine and Romidepsin Are Highly Effective in Patients with PTCL: A Multicenter Phase 2 Study. Blood.

[88] Gordon M.J., Miljkovic M.D., Ng S., Lakhotia R., Melani C., Conlon K., Phelan J.D., Bryant B., Yee L.M., Pittaluga S. (2024). Romidepsin, Azacitidine, Dexamethasone, and Lenalidomide (RAdR) for Relapsed/Refractory T-Cell Lymphoma. Blood.

[89] Saeed H., Mediavilla Varela M., Sahakian E., Naqvi M., Mo Q., Zhang L., Makanji R., Zhang Y., Dong N., Isenalumhe L.L. (2024). A Phase I Study of Duvelisib in Combination with Oral Azacitidine (BMS-986345) in Mature T Cell Lymphoma. Blood.

[90] Moskowitz A., Ganesan N., Chang T., Davey T., Hancock H., Smith M., Assini A., Sarmasti L., Miller T., Gibaldi A. (2024). Dual-Targeted Therapy with Ruxolitinib Plus Duvelisib for T-Cell Lymphoma. Blood.

[91] Reneau J.C., Huang Y., Hanel W., Brammer J.E., Chung C., William B. (2024). Final Results of a Phase II Study of Brentuximab Vedotin and Lenalidomide in Relapsed and Refractory T-Cell Lymphomas. Blood.

[92] Wang X., Guo W., Zhao Y., Li J., Bai O. (2024). The Short-Term Efficacy and Safety of Brentuximab Vedotin Chemotherapy Combined with Chidamide in the Treatment of CD30-Positive Peripheral T-Cell Lymphoma. Blood.

[93] Xia C., Li Y., Sun Z., Huang L., Zeng R., He Y., Wang C., Xiao L., Zhou H. (2024). The Efficacy and Safty of Chidamide and Azacitidine Combined with Chemotherapy As a First-Line Treatment in T Follicular Helper Lymphomas: A Comprehensive Retrospective Study. Blood.

[94] Neelapu S.S., Locke F.L., Bartlett N.L., Lekakis L.J., Miklos D.B., Jacobson C.A., Braunschweig I., Oluwole O.O., Siddiqi T., Lin Y. (2017). Axicabtagene Ciloleucel CAR T-Cell Therapy in Refractory Large B-Cell Lymphoma. N. Engl. J. Med..

[95] Abramson J.S., Solomon S.R., Arnason J., Johnston P.B., Glass B., Bachanova V., Ibrahimi S., Mielke S., Mutsaers P., Hernandez-Ilizaliturri F. (2023). Lisocabtagene Maraleucel as Second-Line Therapy for Large B-Cell Lymphoma: Primary Analysis of the Phase 3 TRANSFORM Study. Blood.

[96] Luo L., Zhou X., Zhou L., Liang Z., Yang J., Tu S., Li Y. (2022). Current State of CAR-T Therapy for T-Cell Malignancies. Ther. Adv. Hematol..

[97] Cwynarski K., Iacoboni G., Tholouli E., Menne T.F., Irvine D.A., Balasubramaniam N., Wood L., Shang J., Zhang Y., Basilico S. (2022). First in Human Study of AUTO4, a TRBC1-Targeting CAR T-Cell Therapy in Relapsed/Refractory TRBC1-Positive Peripheral T-Cell Lymphoma. Blood.

